# Carbon dioxide-enhanced metal release from kerogen

**DOI:** 10.1038/s41598-022-19564-z

**Published:** 2022-09-07

**Authors:** Tuan A. Ho, Yifeng Wang

**Affiliations:** 1 Geochemistry Department, Sandia National Laboratories, Albuquerque, NM 87185 USA; 2 Nuclear Waste Disposal Research and Analysis Department, Sandia National Laboratories, Albuquerque, NM 87185 USA

**Keywords:** Environmental chemistry, Theoretical chemistry

## Abstract

Heavy metals released from kerogen to produced water during oil/gas extraction have caused major enviromental concerns. To curtail water usage and production in an operation and to use the same process for carbon sequestration, supercritical CO_2_ (scCO_2_) has been suggested as a fracking fluid or an oil/gas recovery agent. It has been shown previously that injection of scCO_2_ into a reservoir may cause several chemical and physical changes to the reservoir properties including pore surface wettability, gas sorption capacity, and transport properties. Using molecular dynamics simulations, we here demonstrate that injection of scCO_2_ might lead to desorption of physically adsorbed metals from kerogen structures. This process on one hand may impact the quality of produced water. On the other hand, it may enhance metal recovery if this process is used for in-situ extraction of critical metals from shale or other organic carbon-rich formations such as coal.

## Introduction

Produced water is a major waste product associated with oil and gas extraction. In general, more waste water is produced than oil with a water/oil volume ratio of ~ 3^1^, and every day more than 100 million barrels of produced water^1^ are discharged into the environment^2^. Produced water contains a complex mixture of metals, salts, total dissolved solid, hydrocarbons, and chemical additives used during stimulation and extraction processes. The chemical composition of produced water varies depending on geographic locations, geochemistry of formations, extraction methods, and reservior types (conventional vs. unconventional)^3^. Many metals in produced water are toxic and cause a major environmental problem, especially naturally occurring radioactive materials including ^232^Th, ^238^U, ^226^Ra, ^210^Pb, and ^137^Cs^4^. Due to the complexity of metal partition between geological materials (kerogen and minerals) and fluids, it remains challenge to quantify the source of metals in produced water and develop a strategy to minimize the amount of toxic metals released into produced water.

Kerogen is the largest organic pool on earth. Kerogen is responsible for oil and gas generation, storage, and transport. Numerous molecular studies have focused on CO_2_, CH_4_, and water adsorption on kerogen^5,6^, the associated chemo-mechanical coupling (e.g., swelling)^7,8^, self-diffusion of gas in kerogen matrix^9,10^, and oil/gas flow through kerogen matrix or nanoscale slits^11–13^. However, molecular level understanding of metal adsorption onto kerogen remains elusive. Kerogen is known to concentrate heavy metals due to its high affinity for metal adsorption and complexation^14^. For example, Mo is found to be trapped in sulfur-rich organic matter^15^. As, Cd, Cr, Co, Cu, Fe, Mn, Ni, Pb, and V are found in Niger delta kerogen^16^. Ni, Mo, Ti, and Cr are generally associated with organic matter (e.g., humic acid) in Australian deposits and New Albany Shale of Indiana^17,18^. Similarly, in engineered materials^19^, such as zeolitic imidazolate framework (ZIF-8)^20^ and zinc imidazole salicylaldoxime supramolecule (ZIOS)^21^, chemical bonding of metals (e.g., Cu, Zn) with O and N atoms is explored for capturing metals from aqueous solutions. In addition, drilling and completing fluids in oil and gas industry may contain metal compounds, e.g., cesium formate^22^, barite (BaSO_4_) with trace Zn, Cu, Hg, Fe, Cd, and Cr metals^23^. These compounds may interact with kerogen during an operation.

The association of metals with kerogen can be categorized into two groups: (1) metals/metal clusters deeply embedded in kerogen structures and (2) metals adsorbed on kerogen surfaces (or pore surfaces). In this work, we will focus on the latter, because they are more liable to release upon a change in solution chemistry and therefore, to a larger extent, affect the dissolved metal concentrations in produced water. Unfortunately, the adsorption of metal ions onto porous kerogen surfaces is poorly characterized. Such adsorption is facilitated by the significant presence of aqueous solution in kerogen nanopores^24^. The imbibition of aqueous solution into porous kerogen structure depends on kerogen hydrophobicity^25^ and kerogen can be a hydrophilic material^26,27^. Kerogen maturation reduces H/C and O/C ratios over time and therefore increases the hydrophobicity of the material^28,29^. It is thus of interest to study a possible effect of kerogen maturity on metal adsorption in oil/gas reservoirs.

To curtail the amount of water used in hydraulic fracturing and the amount of water produced in an operation, as well as to use the same stimulation process for subsurface carbon sequestration, supercritical CO_2_ (scCO_2_) has been proposed as a fracking fluid or an enhanced oil/gas recovery agent. Upon injection, scCO_2_ adsorbs onto kerogen structures and displaces CH_4_ and oil^30^. The adsorbed scCO_2_ remains locked in nanoporous kerogen structures. Many studies have demonstrated that injected scCO_2_ may cause dramatic changes in wettability of kerogen^26,31^. In this communication, we will investigate how the scCO_2_ injected would affect metal adsorption on kerogen surfaces. We will conduct molecular dynamics simulations for the metal adsorption on overmature and top of the oil window kerogen (type IID and IIB, respectively)^32^ in the presence or absence of scCO_2_. We will show that injection of scCO_2_ may greatly enhance the release of adsorbed metals from kerogen surface. The work presented below will provide the first assessment of the impact of scCO_2_ on the ion adsorption on kerogen and highlight the importance of kerogen-metal interactions in controlling the quality of produced water and the efficiency of potential in-situ extraction of critical metals from shale or other organic carbon-rich formations such as coal. Different from the current research theme related to kerogen, which focuses mainly on oil/gas adsorption and transport, the work presented will emphasize metal-kerogen interactions under an influence of scCO_2_.

## Method

**Figure 1:**
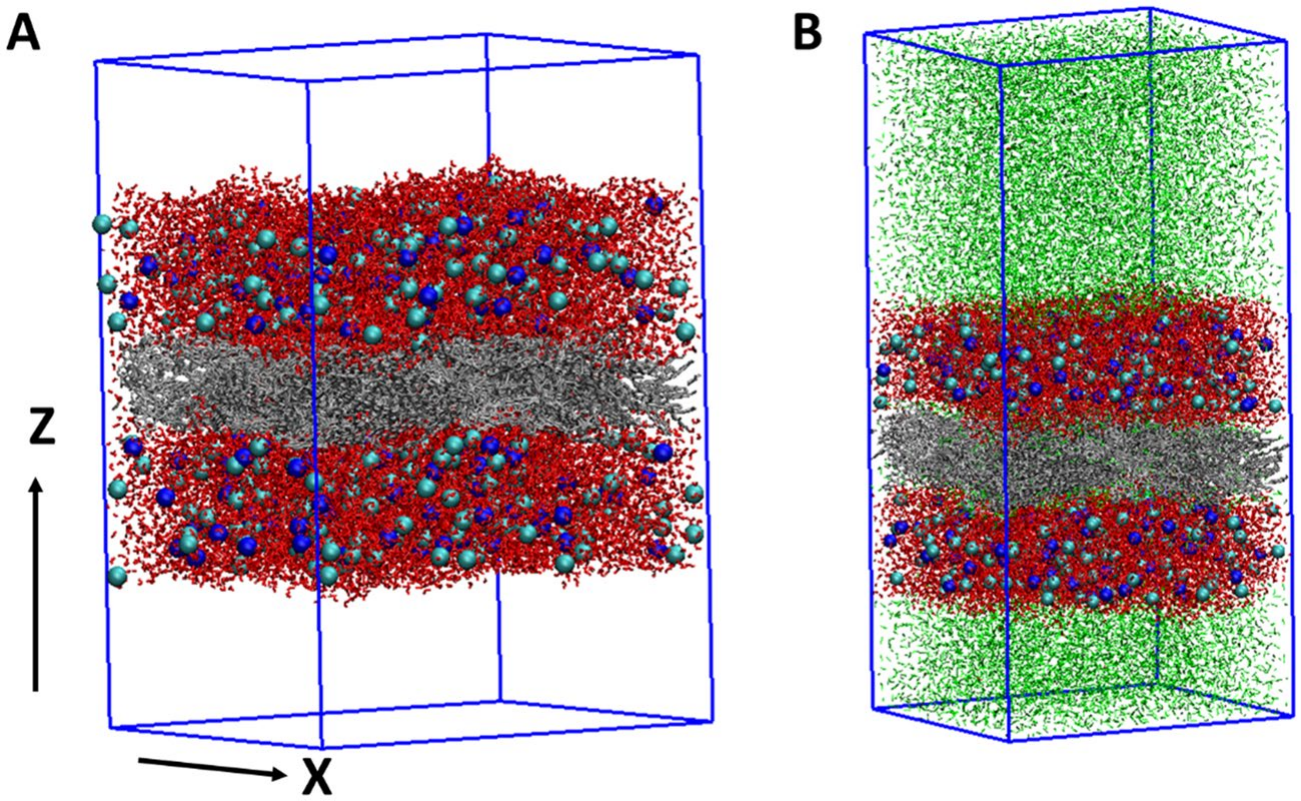
Simulation snapshots illustrating IID-CuCl_2_ (**A**) and IID-CuCl_2_-CO_2_ (**B**) model systems (see Table 1). Color codes: kerogen—silver, water—red, Cu^2+^—blue, Cl^−^—cyan, and CO_2_—green. Simulation box size and number of molecules simulated for each system are reported in Table 1. Some water and CO_2_ molecules can adsorb deeply inside the kerogen porous structure. However, no ion is observed inside the structure due to small pore size.

Simulation snapshots provided in Fig. 1 illustrate the model setup for simulating Cu^2+^, Cs^+^, Cl^−^, and OH^−^ adsorption onto a porous kerogen surface in the presence or absence of scCO_2_. Cu^2+^ and Cs^+^ ions were selected to represent common metal cations found in produced water^4^. Overmature and top of the oil window kerogen structural models (type IID and IIB, respectively)^32^ were used in our simulations. The chemical formulas for kerogen IIB and IID are C_234_H_263_O_14_N_5_S_2_ and C_175_H_102_O_9_N_4_S_2_, respectively. The kerogen surfaces in Fig. 1 were constructed in our previous work^14^. There are –OH functional groups in kerogen. However, for simplicity, no protonation/deprotonation would be allowed in the simulation (i.e., the kerogen surfaces remained to be charge neutral). No information is available on surface protonation/deprotonation of kerogen. The point of zero charge of other alike natural carbon materials such as algae and coal charcoals were found to be close to neutral pH^33^. Therefore, the assumption of no surface protonation/deprotonation may be a reasobale approximation of an actual system. The composition, number of molecules, and simulation box size for all simulations are reported in Table 1. Water molecules were initially placed near the surfaces and ions were randomly distributed in water. For the systems without scCO_2_ (Fig. 1A), simulations were conducted in the NVT (constant number of atoms, volume, and temperature) ensemble, with a vacuum volume in the simulation box. CO_2_ molecules were then filled in the vacuum volume to create the systems with scCO_2_ (Fig. 1B) to study the effect of scCO_2_ on ion adsorption. With the presence of scCO_2_, the simulations were run in the NPT (constant number of atoms, pressure, and temperature) ensemble with a 200 atm pressure imposed in the z dimension. The temperature was set at 300 K for all simulations. The temperature and pressure were controlled using the Nose–Hoover thermostat^34,35^. All systems were simulated until an equilibrium condition reaches (e.g., the number of ions adsorbed on a surface is constant). Accordingly, the simulations without CO_2_ were run for 35 ns, while the simulations with CO_2_ were run for 60 ns to 90 ns.

**Table 1:** Simulation box size and number of molecules in each simulation system. In the IID-Cu(OH)Cl-CO_2_ system, kerogen type IID is simulated with Cu^2+^, OH^−^, Cl^−^, CO_2_, and H_2_O. In the IIB-CsCl system, kerogen type IIB is simulated with Cs^+^, Cl^−^, and H_2_O.

System	Box size (Å^3^)	H_2_O	Cu^2+^ or Cs^+^	Cl^−^	OH^−^	CO_2_
IID-CuCl_2_	89.6 × 103.6 × 150	20,328	180	360		
IIB-CuCl_2_	89.6 × 103.6 × 150	18,480	162	324		
IID-CsCl	89.6 × 103.6 × 150	20,328	180	180		
IIB-CsCl	89.6 × 103.6 × 150	18,480	162	162		
IID-Cu(OH)Cl	89.6 × 103.6 × 150	20,120	180	180	180	
IID-CuCl_2_-CO_2_	89.6 × 103.6 × 216.7	20,328	180	360		14,574
IIB-CuCl_2_-CO_2_	89.6 × 103.6 × 170.8	18,480	162	324		10,646
IID-CsCl-CO_2_	89.6 × 103.6 × 212.6	20,328	180	180		14,069
IIB-CsCl-CO_2_	89.6 × 103.6 × 167.6	18,480	162	162		10,190
IID-Cu(OH)Cl-CO_2_	89.6 × 103.6 × 215.9	20,120	180	180	180	14,579

Water molecules were simulated using a flexible SPC water model^36^. Cu^2+^ ion parameters were taken from Babu and Lim^37^, which accurately reproduce hydration energies. Cs^+^ and Cl^−^ ions were described using Smith and Dang models^38,39^. Lennard–Jones (LJ) parameters for OH^−^ ions are similar to those of a SPC water model, and O charge is − 1.41e and H charge is 0.41e^40,41^. CO_2_ molecules were modeled using the TRaPPE force field^42^. The rigidity of a CO_2_ molecule was maintained by using the algorithm proposed by Kamberaj^43^. The CVFF force field^44^ was used for kerogen (a LAMMPS^45^ data file containing all force field parameters for the kerogen molecule IID can be found in our previous paper^30^). The pairwise LJ potential energy was expressed as: $V_{LJ} = 4\varepsilon \left[ {\left( {\frac{\sigma }{r}} \right)^{12} - \left( {\frac{\sigma }{r}} \right)^{6} } \right],$ where *r* is the distance between two atoms, ε and σ are the depth of the potential energy well and the distance at which the LJ potential is zero, respectively. LJ interactions among atoms were calculated using the Lorentz-Berthelot mixing rules $\varepsilon_{ij} = \sqrt {\varepsilon_{ii} \varepsilon_{jj} }$ and $\sigma_{ij} = (\sigma_{ii} + \sigma_{jj} )/2$. Short range interactions were calculated using a cut-off distance of 10 Å. Long range electrostatic interactions were computed using the PPPM (particle–particle-particle-mesh) solver^46^. All simulations were conducted using the LAMMPS code^45^.

## Results

### Metal adsorption on kerogen surfaces

**Figure 2:**
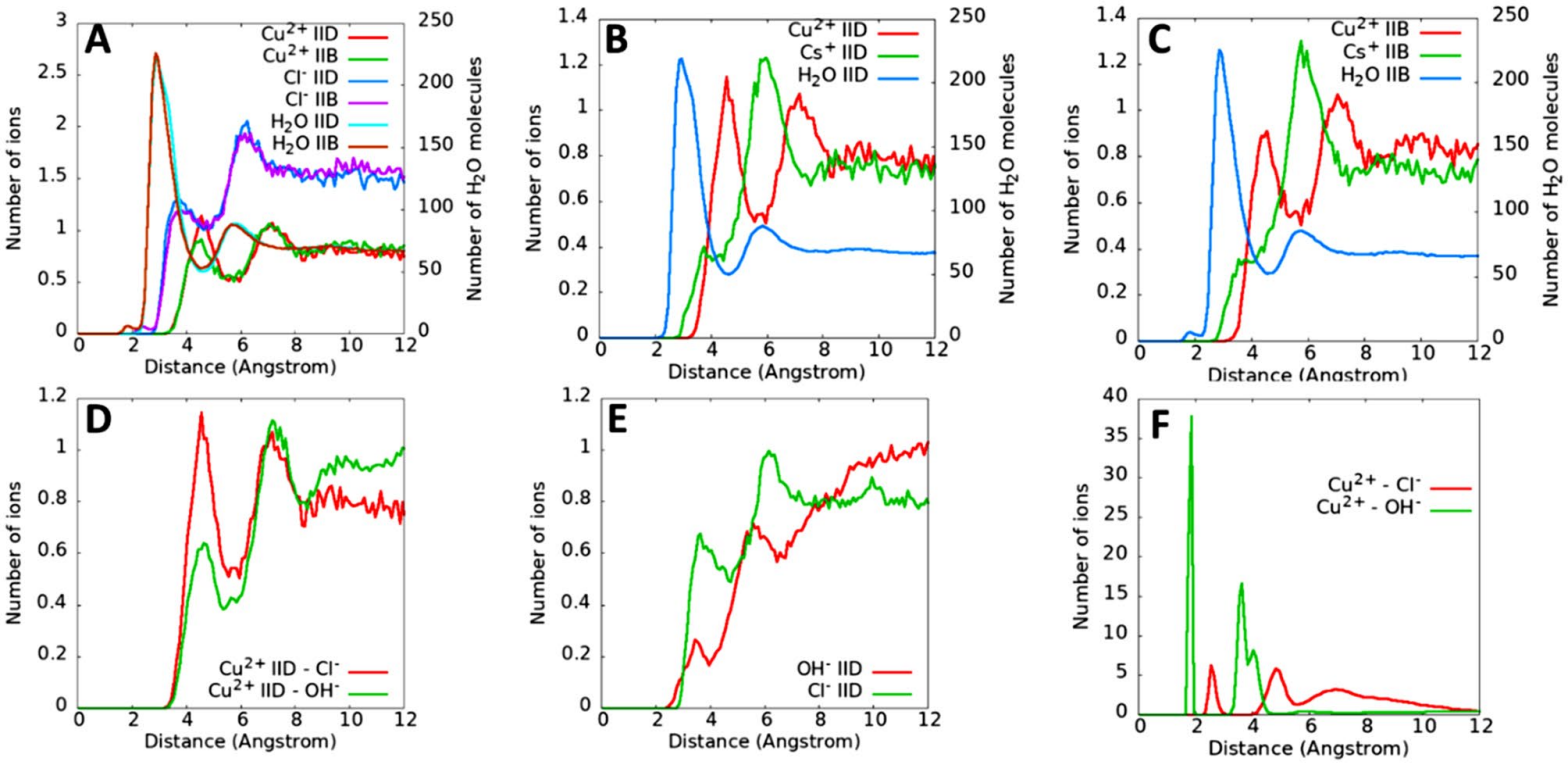
Number of ions (Cu^2+^ and Cl^−^) and water molecules as a function of the distance to the closest kerogen atoms (**A**). Comparison of Cu^2+^ and Cs^+^ adsorption between kerogen IID (**B**) and IIB (**C**) surfaces. Comparison of Cu^2+^ ion adsorption for the systems with and without OH^−^ ions, i.e., for IID-CuCl_2_ (red) and IID-Cu(OH)Cl (green) systems (**D**). Comparison of OH^−^ and Cl^−^ ion adsorption on kerogen IID obtained for IID-Cu(OH)Cl system (**E**). Cu^2+^–Cl^−^ and Cu^2+^–OH^−^ paring calculated from IID-Cu(OH)Cl system (**F**).

Figure 2A reports the number of Cu^2+^ and Cl^−^ ions and water molecules as a function of distance to the closest kerogen atoms. These data are obtained for the IID-CuCl_2_ and IIB-CuCl_2_ systems (Table 1). Because the kerogen surface is very rough^31^, the profile of the number of each species from the closest kerogen atoms (instead of the density profile) is the appropriate selection to quantify the adsorption. The results indicate that Cu^2+^ ions prefer to adsorb as outer sphere complexes (the first Cu^2+^ peak locates at ~ 4.5 Å away from kerogen atoms, between the first and second water peaks, and the second Cu^2+^ peak locates at ~ 7.1 Å away from kerogen atoms, beyond the second water peak). The adsorption of Cu^2+^ ions depends on the interactions of Cu^2+^ ions with water molecules and with kerogen surfaces. Because the kerogen surface is charge neutral, we expect weak interactions of Cu^2+^ ions with surface atoms (dominated by C and H atoms). Therefore, Cu^2+^ adsorption is mainly controlled by its high hydration energy (− 480.4 kcal/mol^47^) that makes it difficult to strip water molecules from the hydration shell to form an inner sphere complex, thus different from its inner-sphere adsorption on silica and alumina surfaces (inner spheres)^48,49^.

**Figure 3:**
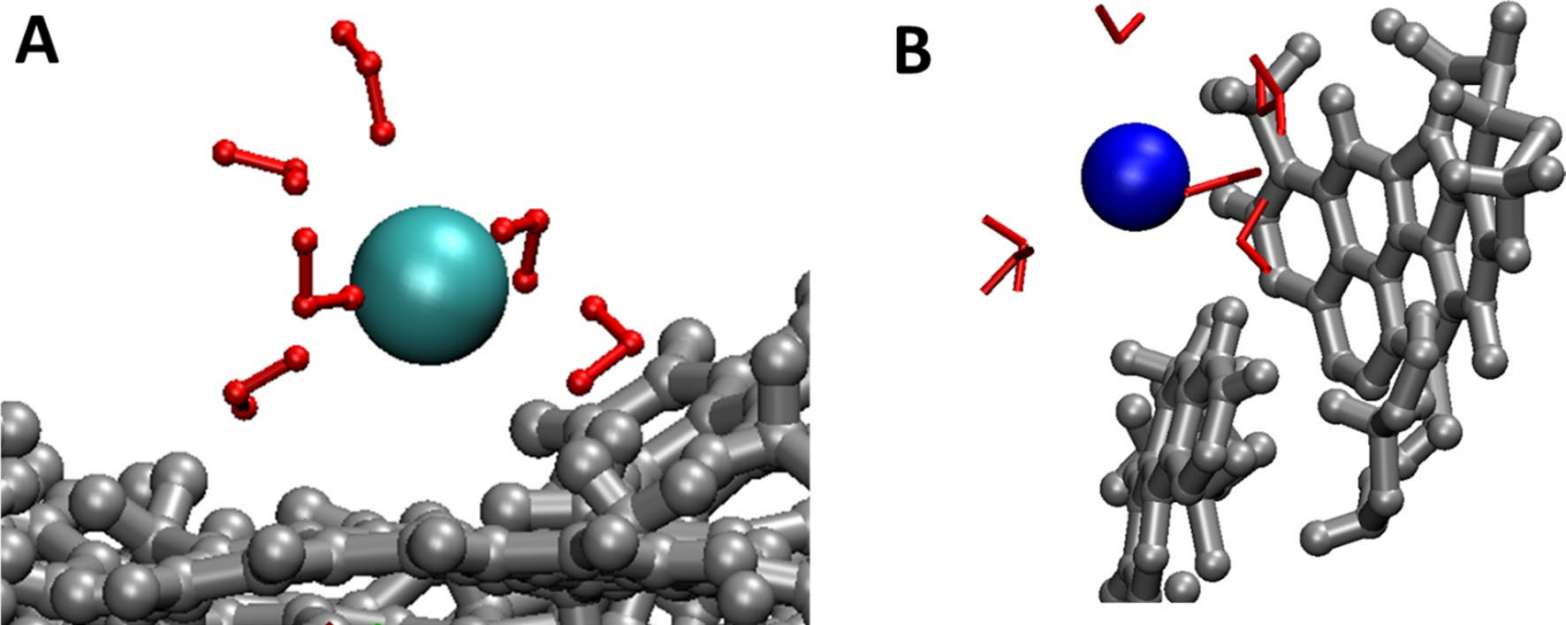
Simulation snapshots demonstrating the inner sphere complexes of Cl^−^ (**A**) and Cs^+^ (**B**) on kerogen IID surface. See Fig. 1 for the color codes.

The results in Fig. 2A also suggest that some Cl^−^ ions adsorb closer to kerogen atoms, i.e., as inner sphere complexes (Fig. 3A), as indicated by the first Cl^−^ peak locating at 3.75 Å away from kerogen atoms, closer than that for Cu^2+^ ions, which is consistent with the lower hydration energy of Cl^−^ (− 81.2 kcal/mol)^47^. However, the majortiy of Cl^−^ ions adsorb still as outer-sphere complexes as the predominant Cl^−^ peak locates at 6.2 Å away from the kerogen surface. The results in Fig. 2A also indicate that there is not any significant difference in the ion adsorption between kerogen IIB and kerogen IID. Compared with kerogen IID, kerogen IIB is less matured and has more funtional groups (e.g., higher O/C, S/C, and N/C ratios)^32^. Note that these ratios are generally small (e.g., 0.1 for O/C)^28^, and thus the atoms that an adsorbed ion can “see” on the kerogen surface are mainly C and H. This may be the reason why we do not observe a significant effect of kerogen maturity on ion physical adsorption.

In Fig. 2B, C, we compare Cu^2+^ and Cs^+^ adsorption on kerogen IID and IIB surfaces. The results for Cs^+^ ions are obtained for the IID-CsCl and IIB-CsCl systems (Table 1). We observe a low intensity Cs^+^ peak at 3.75 Å away from the kerogen atoms, suggesting inner sphere adsorption (see a snapshot in Fig. 3B). The Cs^+^ hydration energy is about − 60 kcal/mol^47^, much smaller than that of Cu^2+^ (− 480.4 kcal/mol), thus making it easier to strip water molecules to form inner sphere complexes. However, since Cs^+^ ions weakly interact with the neutral kerogen surface, the majority of Cs^+^ ions prefer to locate at the same position of the second water layer on both kerogen IIB and IID. In contrast, Cu^2+^ ions prefer to avoid the dense water layers and adsorb between the first and the second water layers, or beyond the second water layer.

In Fig. 2D we compare Cu^2+^ adsorption onto kerogen IID surface from solutions with or without OH^−^ ions for the IID-CuCl_2_ and IID-Cu(OH)Cl systems (Table 1). The results indicate that the first Cu^2+^ peak observed for IID-CuCl_2_ system (red lines) diminishes due to the presence of OH^−^ ions (green lines). Because of very limited amount of OH^−^ ions found near the kerogen surface, compared to Cl^−^ ions (Fig. 2E), and because of Cu^2+^–OH^−^ ions paring (Fig. 2F, i.e., more Cu^2+^ ions pair with OH^−^ ions than with Cl^−^ ions), the adsorption of Cu^2+^ ions can be considered as the adsorption of Cu^2+^–OH^−^ pairs. These complexes affect the amount of Cu^2+^ ions accumulate near the surfaces (e.g., the first Cu^2+^ peak), but do not affect Cu^2+^ accumulation far away from the surface (second Cu^2+^ peak).

### Metal adsorption on kerogen surface in scCO_2_

**Figure 4:**
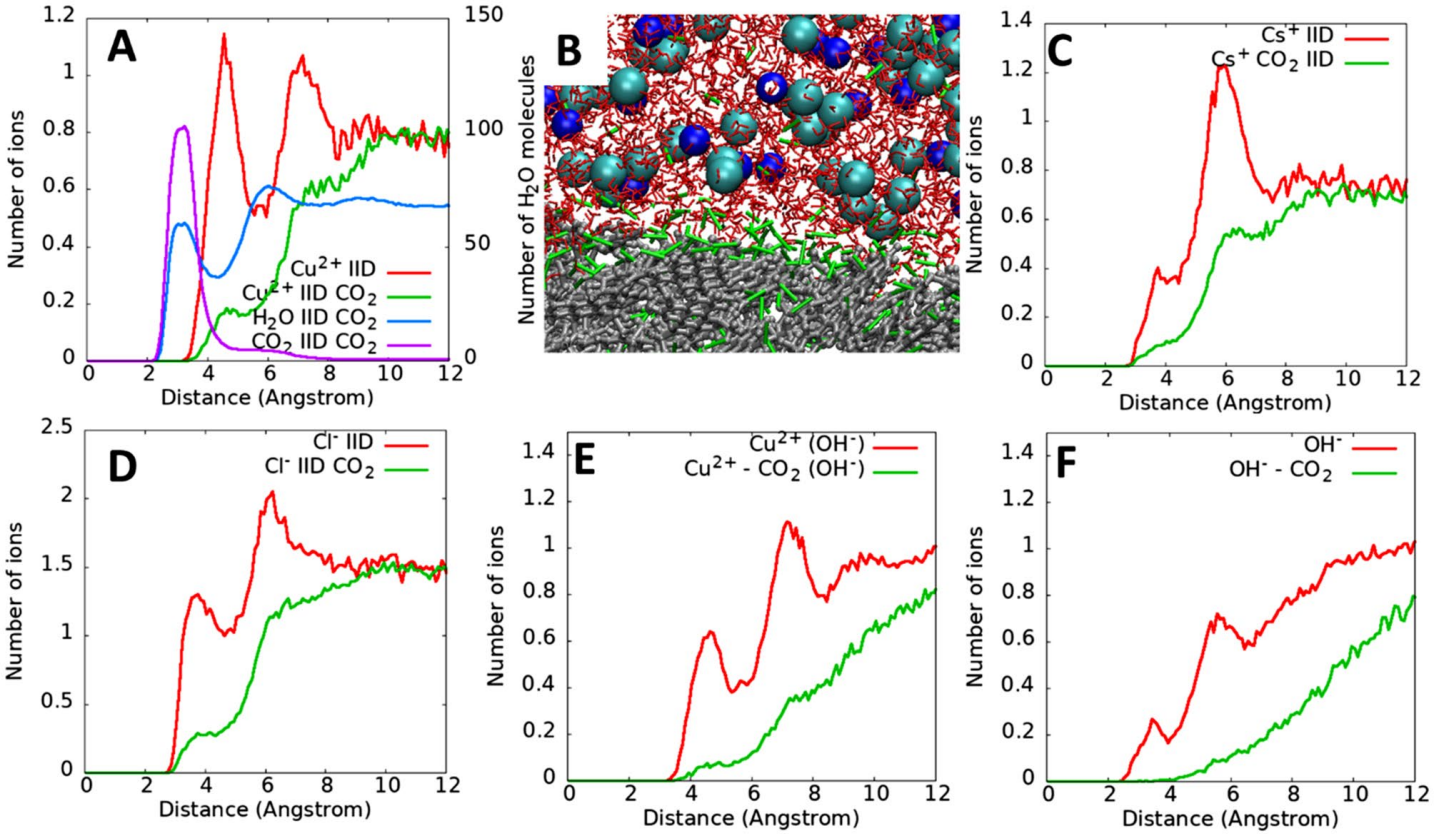
Number of Cu^2+^ as a function of distance from the closest kerogen IID surface atoms for the IID-CuCl_2_ (red line) and IID-IID-CuCl_2_-CO_2_ (green line) systems (**A**). Distributions of water and CO_2_ molecules are also shown for the IID-CuCl_2_-CO_2_ system. The simulation snapshot demonstrates the adsorption of CO_2_ (green) on kerogen (silver) in aqueous solution (water: red, Cu^2+^: blue, Cl^−^: cyan) (**B**). Distribution of Cs^+^ (**C**) and Cl^−^ (**D**) ions on kerogen IID in the presence/absence of CO_2_. Distribution of Cu^2+^ (**C**) and OH^−^ (**D**) ions on kerogen IID in the presence/absence of CO_2_ for IID-Cu(OH)Cl-CO_2_ system.

In Fig. 4A we report the results for IID-CuCl_2_-CO_2_ system (Table 1) to eluciate the effect of supercritical CO_2_ on ion adsorption. Note that CO_2_ molecules are initially added to the vacuum space in Fig. 1A. During the simulation CO_2_ molecules diffuse through water and adsorb onto kerogen structure (Figs. 1B, 4B). The results indicate that after CO_2_ is added, the first and second Cu^2+^ peaks for the system without CO_2_ (i.e., IID-CuCl_2_ system) diminish (red vs. green lines), suggesting that Cu^2+^ ions desorb from the kerogen atoms. When Cu^2+^ ions within 6 Å from the kerogen atoms (i.e., the first minimum on the red line, Fig. 4A) are considered, about 78% of the adsorbed cations desorbs from kerogen in the presence of scCO_2_. When Cu^2+^ ions within 8.2 Å from kerogen atoms (i.e., the second miniumum on the red line, Fig. 4A) are considered, about 60% of the cations desorbs after scCO_2_ is introduced. In other words, injection of scCO_2_ causes the adsorbed Cu^2+^ ions to desorb from kerogen surfaces. The adsorption of scCO_2_ on kerogen surface is indicated by a CO_2_ peak at 3 Å away from kerogen atoms (purple line, Fig. 4A). The purple profile for CO_2_ also demonstrates the formation of a monolayer of CO_2_ on a kerogen surface and a futher decrease in the number of CO_2_ away from the surface due to the limited CO_2_ solubility in water. When CO_2_ molecules accumulate near the surface, they partly replace water molecules, leading to the lower intensity water peak (blue line, Fig. 4A vs. blue line, Fig. 2A) and desorption of adsorbed ions (Fig. 4C-F).

The desorption of water from kerogen surface due to scCO_2_ adsorption was initially reported in our previous work^31^. The adsorbed layer of scCO_2_ between water and kerogen surfaces acting like a lubricant to facilitate water flow on the kerogen surfaces. The main reason for a CO_2_ molecule substitution for a H_2_O molecule to adsorb on the surface is because CO_2_ interacts with kerogen surface more strongly than H_2_O (− 6.2 kcal/mol for CO_2_ vs. − 4.7 kcal/mol for water)^26^. The adsorbtion of CO_2_ also causes the change in wettability of kerogen (i.e., increases hydrophobicity)^26^. These phenomena were computationally confirmed by other groups^50^. Note that increasing hydrophobicity of kerogen upon injection of scCO_2_ can enhance water exclusion, and therefore might futher increase water release (and hence heavy metals). Our current work provides the first assessment of the impact of scCO_2_ on the ion adsorption, which requires futher experimental investigation.

## Conclusions

Using molecular dynamics simulations, we investigated ion adsorption on kerogen surface in the presence or absence of scCO_2_. Due to weak interactions of ions with neutral kerogen surfaces, the majority of Cu^2+^, Cs^+^, Cl^−^, and OH^−^ ions adsorb as outer sphere complexes. Some Cs^+^ and Cl^−^ ions adsorb as inner sphere complexes. We also found that the presence of OH^−^ ions reduces the number of Cu^2+^ ions adsorbed due to ion paring. All ions were observed to be desorbed when scCO_2_ was introduced to the system. For the conditions simulated in this work, we observed that about 60% of Cu^2+^, 50% Cs^+^, and 55% Cl^−^ within ~ 8 Å from the kerogen atoms desorb when introducing scCO_2_ into the system*.* This process on one hand may impact the quality of produced water. On the other hand, it may enhance metal recovery if this process is used for in-situ critical metal extraction from shale or other organic carbon-rich formations such as coal. The work presented here can be extended and validated through adsorption and leaching experiments as well as by quantum-based calculations to further determine the kinetics and thermodynamics of metal adsorption onto kerogen under various scCO_2_ pressure, environmental temperature, and kerogen maturity.

## Data Availability

The datasets used and/or analyzed during the current study available from the corresponding author on reasonable request.
